# Alpha-linolenic acid reshapes the gastrointestinal ecology to enhance immune function in dairy cows: insights from microbiome and metabolome profiling

**DOI:** 10.3389/fmicb.2025.1687786

**Published:** 2025-11-14

**Authors:** Hongjin Ji, Zhantao Yang, Yangyi Hao, Yajing Wang, Zhijun Cao, Hongjian Yang, Wei Wang, Shengli Li

**Affiliations:** State Key Laboratory of Animal Nutrition and Feeding, College of Animal Science and Technology, China Agricultural University, Beijing, China

**Keywords:** α-linolenic acid, gastrointestinal microbiota, microbial metabolite, inflammatory response, dairy cows

## Abstract

**Introduction:**

Alpha-linolenic acid (ALA), an essential polyunsaturated fatty acid, modulates gastrointestinal microbiota and host immunity, yet its regulatory mechanisms in dairy cows remain unclear.

**Methods:**

This study investigated how dietary ALA influenced gut microbiota, metabolome, and immune function in lactating Holstein cows. Ten cows were randomly assigned to two groups (*n* = 5) receiving either a low-ALA (LALA, 5.02 ± 0.09% ALA of total fatty acids) or high-ALA diet (HALA, 32.04 ± 1.55% ALA of total fatty acids). Rumen fluid, feces, and blood samples were analyzed post-intervention.

**Results:**

The HALA group exhibited increased ruminal abundance of *Eubacterium coprostanoligenes group* and *Ruminococcus* (*p* < 0.05), alongside reduced proinflammatory metabolites including dodecanoic acid, myristic acid, and prostaglandin I_2_ in the rumen. Plasma leukotriene C_4_ levels were also decreased (*p* < 0.05). Metabolomic enrichment analysis revealed significant downregulation of arachidonic acid metabolism. Correlation analyses demonstrated that *Eubacterium coprostanoligenes group* negatively associated with suppressed prostaglandin I_2_ (rumen metabolite) and leukotriene C_4_ (plasma metabolite), but positively correlated with enriched fecal *Clostridia UCG-014* and *Ruminococcus*.

**Discussion:**

These findings indicate that high dietary ALA reshapes gastrointestinal microbiota and attenuates inflammatory responses by inhibiting microbial-metabolite-driven arachidonic acid metabolism, thereby enhancing immune regulation in dairy cows.

## 1 Introduction

Alpha-linolenic acid (ALA, 18:3n-3, cis-9, cis-12, cis-15) is an omega-3 (ω-3) polyunsaturated fatty acid (PUFA) characterized by three cis-configured double bonds (Yuan et al., 2022). It is primarily found in flaxseeds, perilla seeds, algae, and fish (Venkatesh and Muthu, 2025). In recent years, the nutritional and regulatory potential of ALA has garnered increasing attention, particularly for its role in modulating the host's gastrointestinal microecology, alleviating chronic inflammatory responses, and enhancing immune function (Kra et al., 2023; Livingston et al., 2023; Määttänen et al., 2020). Previous studies have shown that ALA not only directly modulates the host's immune response through its involvement in signaling pathways, but also indirectly affects host health by altering the composition and metabolic profiles of the gastrointestinal microbiota (Fu et al., 2021; Samrit et al., 2025; Vargas et al., 2020).

At the microbial level, ALA exerts a significant regulatory influence on the gastrointestinal microbiota (Livingston et al., 2023; Wang et al., 2019; Yang et al., 2024). In ruminants, dietary supplementation with flaxseed oil significantly increases the abundance of bacterial families Veillonellaceae and Christensenellaceae in the rumen while modulating ruminal metabolite composition (Wang et al., 2019). High-dose ALA supplementation further reshapes the ruminal microbiota, evidenced by an increased relative abundance of the *Prevotella* and decreased levels of *Butyrivibrio* and *Clostridium* (Yang et al., 2024). In monogastric animal models, ALA enhances intestinal microbial diversity and significantly elevates the abundance of beneficial taxa such as Lachnospiraceae and Bifidobacterium (Livingston et al., 2023), thereby mitigating microbiota dysbiosis induced by parasitic infections or high-fat diets (Yang J. et al., 2023; Zhu L. et al., 2020). These findings collectively suggest that ALA may contribute to maintaining microecological balance by regulating intestinal microbiota diversity and abundance.

From a molecular perspective, ALA-derived metabolites, including 13-hydroxy-octadecadienoic acid (13-OH) and 13-oxo-octadecadienoic acid (13-oxo), promote the differentiation of anti-inflammatory M2 macrophages via activation of the G protein-coupled receptor 40 (GPR40) signaling pathway (Ohue-Kitano et al., 2018). These metabolites also suppress proinflammatory responses by downregulating TNF-α and IL-6 expression through NF-κB and MyD88 signaling pathways (Kra et al., 2023; Zhang et al., 2017; Zhu X. et al., 2020). In disease models, ALA alleviates atherosclerotic progression in ApoE–/– mice (Li et al., 2022), reduces the Firmicutes to Bacteroidetes ratio and arachidonic acid (AA, 20:4n-6) levels in the small intestine of high-fat diet-fed mice, and facilitates the development of an anti-inflammatory phenotype (Todorov et al., 2020). Moreover, ALA attenuates endotoxin levels and peritoneal inflammation in endometriosis (EMS) model mice by restoring gut barrier integrity and modulating microbial composition (Ni et al., 2021), and has shown protective effects in models of acute lung injury (ALI) (Zhu X. et al., 2020), colitis (Reifen et al., 2015), and liver cancer immunotherapy (Liu et al., 2024).

Based on existing evidence from prior research, the potential immunomodulatory mechanism of ALA is closely linked to its modulation of AA metabolism (Norris and Dennis, 2012). AA is a key n-6 PUFA and serves as the primary precursor for bioactive eicosanoids—such as prostaglandins, thromboxanes, and leukotrienes—which are potent signaling molecules involved in inflammation (Hanna and Hafez, 2018). Notably, prostaglandin E_2_ (PGE_2_) and leukotriene B_4_ (LTB_4_) act as central mediators that initiate, amplify, and sustain inflammatory responses by promoting immune cell recruitment and stimulating the production of pro-inflammatory cytokines (Frolov et al., 2013; Patil et al., 2019; Wagner et al., 2020). As an n-3 PUFA, dietary ALA competes with AA for shared metabolic enzymes, including cyclooxygenase (COX) and lipoxygenase (LOX), thereby reducing the synthesis of pro-inflammatory eicosanoids (Czapski et al., 2016; Dennis and Norris, 2015; Dong et al., 2016; Hanna and Hafez, 2018; Hassan et al., 2010). Furthermore, elongation and desaturation of ALA yield longer-chain metabolites such as eicosapentaenoic acid (EPA), which can be converted into specialized pro-resolving mediators (SPMs), including resolvins, that actively suppress inflammation and promote resolvins (Norris and Dennis, 2012). Therefore, we propose that increasing dietary ALA intake may competitively inhibit AA metabolism, shift the eicosanoid profile toward an anti-inflammatory and pro-resolving state, and thus promote a more tightly regulated immune response. This rebalancing of lipid mediator networks may represent a core molecular mechanism underlying ALA-mediated immunomodulation in dairy cows.

Accumulating evidence supports ALA's role in host health regulation via the “microbiota-immune axis” across various disease models (Gao et al., 2020; Yang et al., 2021a,b; Yang C. et al., 2023; Zhang et al., 2017; Zhu L. et al., 2020). However, most studies have been limited to mice, with investigations specifically addressing ruminants, particularly dairy cows, remaining limited. Therefore, it remains largely unclear whether ALA can modulate immune processes and overall health in dairy cows through the regulation of gastrointestinal microbiota and associated metabolic functions. In this study, we analyzed the microbial community in the rumen—the primary site of digestion—to elucidate the initial digestive and metabolic processes of ALA. Concurrently, fecal samples were collected and examined to evaluate the composition of the distal gut microbiota. This integrative approach was guided by several key considerations. Following ruminal fermentation, dietary residues and microbial metabolites transit to the hindgut, where they undergo further microbial transformation, yielding numerous bioactive compounds. These hindgut-derived metabolites may enter systemic circulation via the gut-organ axis, particularly the gut-immune axis, thereby exerting significant modulatory effects on the host's systemic immune status (Ni et al., 2017; Zhou et al., 2020). Furthermore, limited research has investigated the impact of varying dietary ALA proportions on the fecal microbiome. Hence, a comprehensive assessment of both ruminal and fecal microbial communities enables a more complete understanding of how dietary ALA shapes the gastrointestinal microbiota along the entire digestive tract and illuminates potential pathways underlying its systemic immunomodulatory effects. Clarifying this issue holds both theoretical value for understanding novel immune regulatory mechanisms in ruminants and practical implications for developing sustainable nutritional strategies and improving dairy cow health management. Based on this background, we hypothesized that ALA might regulate the host's immune function by altering the composition of the gastrointestinal microbiota in dairy cows.

## 2 Materials and methods

### 2.1 Animal management and sample collection

The experiment was implemented at a commercial dairy farm located in Shunyi District, Beijing, China. All procedures involving animals were authorized by the Ethics Committee of the School of Animal Science and Technology, China Agricultural University (Approval No. AW61902202-1-3). Ten lactating multiparous Holstein dairy cows were selected and randomly assigned to two experimental groups using a computer-generated random number sequence. Prior to the initiation of the experiment, no significant differences were observed between the groups in terms of daily milk yield (LALA: 30.4 ± 3.78 kg/d; HALA: 31.0 ± 2.92 kg/d), days in lactation (LALA: 209.6 ± 11.39 d; HALA: 209.6 ± 10.88 d), or parity (all second parity), indicating successful randomization and baseline comparability. Cows in each group received one of two distinct diets: a low ALA diet (LALA) containing 5.02 ± 0.09% ALA of total fatty acids or a high ALA diet (HALA) containing 32.04 ± 1.55% ALA of total fatty acids replacing the fat powder (Wilmar Oil Technology Co., Ltd., Tianjin, China; C16:0 > 90%) with an equal amount of fat from extruded flaxseed in the total mixed ration (TMR). Both diets were designed to have equivalent fat content (59.5 ± 0.1 g/kg dry matter [DM]) and equal energy levels (7.60 ± 0.09 MJ net energy for lactation/kg DM), with matched crude protein (CP) concentrations of 159.3 ± 0.28 g/kg DM. Nutritional equivalence was ensured by modifying the inclusion rates of soybean meal, corn, and soybean hulls in the HALA formulation while adhering to NRC (National Research Council, 2001) guidelines for lactating cows. The experimental period lasted 21 days, with milking conducted at 07:00, 14:00, and 19:00 each day, followed by TMR feeding at 07:30 and 14:30. All cows had *ad libitum* access to feed and water. The components, nutritional contents and fatty acid compositions of these two diets were consistent with those described in the published article by Yang et al. (2024).

### 2.2 Apparent nutrient digestibility

To investigate the apparent digestibility of nutrients, feed samples were collected twice weekly. Fecal samples were collected on day 19 (00:00, 09:00, 18:00), day 20 (03:00, 12:00, 21:00), and day 21 (06:00, 15:00) of the experiment, totaling eight collections. Additionally, fecal samples collected from each cow at 06:00 on day 21 were immediately aliquoted into 2-mL cryovials and stored at −80 °C for subsequent microbial analysis. The collection of fecal samples was aimed at evaluating the impact of dietary ALA intervention on the microbial composition in the distal intestine of dairy cows, with the objective of investigating potential associations between hindgut fermentation characteristics and host immune parameters. Subsequently, the fecal samples from each cow were thoroughly homogenized, and approximately 400 g of the pooled sample was collected. To stabilize nitrogen content, 10% tartaric acid was added at a ratio of 25% of the fecal weight. The nutrient compositions of TMR and fecal samples were determined according to a previously described method (Guo et al., 2022) including DM, organic matter (OM), crude protein (CP), ether extract (EE), neutral detergent fiber (NDF), and acid detergent fiber (ADF). Acid-insoluble ash (AIA) was employed as an endogenous indicator to evaluate the apparent total tract digestibility (ATTD) of diets (Van Keulen and Young, 1977). The calculation formula was presented as follows:


$$\begin{array}{l} ATTD=100\times \left(1-\frac{A\times D}{B\times C}\right) \end{array}$$

In the formula, A represents the concentration of AIA in the diet (g/kg); B represents the concentration of the nutrient in the feed (g/kg); C represents to the concentration of AIA in the feces (g/kg); D represents to the concentration of the nutrient in the feces (g/kg).

### 2.3 Fatty acid composition in the rumen

On Day 21, rumen fluid samples were collected before morning feeding using a transoral stomach tube. The initial 50 mL of collected fluid was discarded to minimize contamination from saliva. The collected samples were preserved as a heterogeneous solid-liquid mixture containing feed particles and microbial components, without undergoing filtration or centrifugation. This approach was designed to rapidly capture the initial and most sensitive alterations in fatty acid profiles within the rumen contents—following dietary supplementation with ALA, by direct analysis of the whole mixture. The remaining rumen fluid was immediately transferred into four 2-mL cryovials and stored at −80 °C for subsequent fatty acid analysis. To analyse fatty acid concentration in rumen fluid, a 100 μL aliquot of rumen fluid was transferred to a 15 mL screw-cap glass tube. Subsequently, 4 mL of acetyl chloride–methanol reagent (1:10, v/v), 1 mL of C11:0 internal standard solution (1 mg/mL), and 1 mL of n-hexane were added sequentially. The tube was tightly capped and vortexed thoroughly to ensure homogenization. Methylation of fatty acids was carried out by incubating the mixture at 80 °C in a water bath for 2 hours. Following the reaction, the tube was cooled to room temperature, after which 5 mL of 7% (w/v) potassium carbonate solution was added to neutralize excess acid. The mixture was vortexed again and centrifuged at 1,200 r/min for 5 min. The upper organic layer was collected and filtered through a 0.2 μm microporous membrane filter, and the filtrate was transferred to a sample vial for analysis. Fatty acid methyl esters (FAMEs) were analyzed using an Agilent 6890 gas chromatograph (Agilent, USA) equipped with a flame ionization detector (FID). Chromatographic separation was achieved on a DB-23 capillary column (60.0 m × 250 μm × 0.25 μm, Agilent J&W, USA). The injector and FID temperatures were set at 260 °C and 270 °C, respectively. High-purity helium was used as the carrier gas at a constant flow rate of 2.0 mL/min. A 1 μL sample was injected in split mode with a split ratio of 30:1. The concentrations of individual fatty acids were quantified based on the ratio of their peak areas relative to that of the C11:0 internal standard. To bridge the alterations observed in the upper gastrointestinal tract with ultimate production outcomes, we have referenced our team's prior study conducted on the same animal model (Yang et al., 2024) in the Discussion section. That work systematically demonstrated the significant effects of the identical dietary intervention on milk fatty acid composition.

### 2.4 Serum biochemical indexes

Blood samples were collected from the tail root vein before morning feeding on the last day of the experiment. Whole blood was collected into both non-anticoagulant tubes and EDTA-anticoagulant tubes, with each tube containing approximately 10 mL of blood. Immediately after collection, the blood samples were centrifuged at 4,000 r/min for 15 min. The resulting supernatant was transferred into 2-mL cryovials and promptly stored at −80 °C for further analysis. Serum samples were analyzed using a Hitachi 7600 automatic biochemical analyzer (Hitachi Co., Ltd., Tokyo, Japan). Concentrations of glucose (GLU), total triglycerides (TG), total cholesterol (TC), blood urea nitrogen (BUN), superoxide dismutase (SOD), total antioxidant capacity (T-AOC), glutathione peroxidase (GSH-Px), total protein (TP), albumin (ALB), globulin (GLB), alanine aminotransferase (ALT), aspartate aminotransferase (AST), alkaline phosphatase (ALP), and total bilirubin (T-BIL) were detected. Insulin (INS) content was detected by radioimmunoassay using a Champion radioimmunoassay counter (BFM-96, Zhongcheng Electromechanical Technology Co., Ltd., Hefei, China). Non-esterified fatty acids (NEFA) and β-hydroxybutyric acid (BHBA) contents were measured using commercial ELISA kits according to the manufacturer's instructions (Nanjing Jiancheng Bioengineering Institute, Nanjing, China).

### 2.5 DNA extraction, 16S rRNA gene amplification, and high-throughput sequencing of rumen fluid and fecal samples

Total DNA from rumen bacteria was extracted using a FastDNA^®^ SPIN for the soil kit (MP Biomedicals, Solon, USA) following the manufacturer's protocols and fecal bacterial DNA was isolated with a DNeasy PowerSoil Kit (cat. no. 47014, Qiagen, Hilden, Germany) according to the provided instructions (Hao et al., 2020). The concentration and purity of the extracted and isolated DNA was determined using a NanoDrop 2000 UV-Vis spectrophotometer (Thermo Scientific, Wilmington, DE, USA). Polymerase chain reaction (PCR) reaction systems were configured using qualified genomic DNA samples and corresponding fusion primers for amplification. The amplification primers were 338-F (5′-ACTCCTACGGGAGGCAGCA-3′) and 806-R (5′-GGACTACHVGGGTWTCTAAT-3′). PCR amplification products were purified using BECKMAN AMPure XP Beads and Agencourt AMPure XP Beads, dissolved in elution buffer, labeled, and used for library construction. The fragment range and concentration of the library were detected using an Agilent Bioanalyzer. Qualified libraries were sequenced in paired-end on the NovaSeq PE250 platform and Illumina MiSeq PE300 platform respectively based on the size of the insert fragment. Raw sequence data were deposited in the Sequence Read Archive (SRA) of the NCBI under accession number PRJNA1295766 and PRJNA1295848. Microbiome bioinformatics analyses were conducted using QIIME2 (version 2019.4) as previously described (Wang et al., 2025). Briefly, cutadapt plugin was used to trim adapter and primer sequences from the raw reads, removing potential contamination and preserving the target region for further analysis (Caporaso et al., 2010). Subsequently, the DADA2 plugin in QIIME2 was employed to perform quality control, denoising, chimera removal, and amplicon sequence variant (ASV) inference with single-nucleotide resolution (Callahan et al., 2016). ASVs were inferred based on exact sequence matching at a 100% similarity threshold. Taxonomic assignments for each ASV were carried out using a Naive Bayes classification model trained on the SILVA reference database.

### 2.6 Detection of rumen fluid metabolome and plasma metabolome

Rumen fluid (100 μL) was mixed with 400 μL extraction solvent (acetonitrile-methanol, 1:1) by vortexing in 2-mL tubes, sonicated for 10 min, incubated at −20 °C for 1 h, then centrifuged (13,000 rpm, 15 min, 4 °C). The supernatant was re-centrifuged under identical conditions. Finally, 50 μL supernatant was analyzed by Liquid Chromatography-Mass Spectrometry (LC-MS, Waters, UPLC; Thermo, Q Exactive) using a Waters Acquity UHPLC system with an ACQUITY UPLC BEH Amide column (1.8 μm, 2.1 × 100 mm). Plasma metabolomics profiling methodology employed in this study was in accordance with the protocol established by Dai et al. (2025). Briefly, The sample was thawed at 4 °C and vortexed for 1 min to ensure homogeneous mixing. An aliquot was transferred to a 2 mL centrifuge tube, followed by the addition of 400 μL of methanol and vortexing for 1 min. After centrifugation at 12,000 rpm for 10 min at 4 °C, the entire supernatant was transferred to a new 2 mL centrifuge tube and subjected to concentration under vacuum until dryness. The residue was reconstituted in 150 μL of 80% aqueous methanol containing 4 ppm 2-chloro-L-phenylalanine, filtered through a 0.22 μm membrane filter, and transferred to an autosampler vial for LC-MS analysis. Finally, 2.0 μL of the filtrate was injected into a Vanquish UHPLC System (Thermo Fisher Scientific, USA) coupled to a Orbitrap Exploris 120 (Thermo Fisher Scientific, USA) mass spectrometer. Liquid chromatography (LC) separation was performed using an ACQUITY UPLC ^®^ HSS T3 column (1.8 μm, 2.1 × 100 mm) (Waters, Milford, MA, USA). For LC-ESI(+)-MS analysis, the mobile phase consisted of solvent A (0.1% formic acid in acetonitrile, v/v) and solvent B (0.1% formic acid in water, v/v), delivered at a flow rate of 0.30 mL/min. The column was maintained at 40 °C. Chromatographic separation was performed using the following gradient program: 8 % mobile solvent A from 0 to 1 min, 8–98% mobile solvent A from 1 to 8 min, 98 % mobile solvent A from 8 to 10 min, 98–8% mobile solvent A from 10 to 10.1 min, and 8% mobile solvent A from 10.1 to 12 min. For LC-ESI(-)-MS analysis, the mobile phase consisted of solvent C (acetonitrile) and solvent D (ammonium formate, 5 mM), delivered at a flow rate of 0.30 mL/min. The column was maintained at 40 °C. Chromatographic separation was performed using the following gradient program: 8% solvent C from 0 to 1 min, 8–98% solvent C from 1 to 8 min, 98% solvent C from 8 to 10 min, 98–8% solvent C from 10 to 10.1 min, and 8% solvent C from 10.1 to 12 min. Raw data were processed using Compound Discoverer software (Thermo Fisher). Metabolites were identified by accurate mass-to-charge ratio (m/z), retention time, and comparison of MS/MS spectra against the Human Metabolome Database (HMDB). Subsequently, the identified metabolites were annotated using the Kyoto Encyclopedia of Genes and Genomes (KEGG) database for pathway analysis.

Raw mass spectrometry data were converted to mzXML format using ProteoWizard software. Subsequent processing, including retention time adjustment, peak detection, and cross-sample alignment, was performed with the R-based XCMS package (Navarro-Reig et al., 2015). Data quality control involved four sequential steps: retention of features required detection in >50% of experimental samples; missing value imputation using 50% of the minimum detectable abundance; LOESS correction against quality control samples to mitigate instrumental drift (Gagnebin et al., 2017); internal standard normalization followed by exclusion of features exhibiting relative standard deviation (RSD) >30% (Dunn et al., 2011).

### 2.7 Statistical methods

A completely randomized design was used in this study. Differences in ATTD of nutrients, serum biochemical indices and rumen fatty acid content were compared using the Student's *t*-tests of SAS 9.4 (SAS Institute Inc., Cary, NC, USA). Prior to conducting the Student's *t*-tests, all data were assessed for normality using the Shapiro-Wilk test and for homogeneity of variance using Levene's test. The results indicated that all assumptions underlying the Student's *t*-tests were satisfied. The model was presented as follows:

*Y*_*ij*_ = μ + *Trt*_*i*_ + ε_*ij*_

In the formula, where Y_ij_ was the dependent variable; μ was the overall mean; Trt_i_ was the fixed effect of the treatment; ε_ij_ was the random error. Statistical significance was set at *p* < 0.05. Data were presented as mean, with the standard error of the mean (SEM) shown separately. GraphPad Prism 8 (GraphPad Software, San Diego, CA, USA) was used to draw a visual violin diagram of apparent indicators.

Multivariate analysis of the 16S rRNA gene sequencing data was performed using R Studio. Differences of alpha diversity (Chao 1 index, Observed species index, and Shannon index) and beta diversity were compared using the vegan and picante packages. Beta diversity was assessed using principal coordinate analysis (PCoA) based on Bray-Curtis distances. The relative abundance of each microbial phylum and genus were calculated, and histograms were generated to analyze the compositional characteristics of rumen and fecal microbiota in the two groups. Spearman correlation analysis was performed separately for the top 20 rumen bacterial genera and fecal bacterial genera based on relative abundance in the LALA and HALA groups. Correlations with an absolute correlation coefficient |r| > 0.6 and a *p*-value < 0.05 were considered statistically significant. Linear discriminant analysis effect size (LEfSe) was employed to identify differential microbies in the rumen and fecal bacteria of the LALA and HALA groups using the microeco package, with the threshold of linear discriminant analysis (LDA) threshold > 2 and *p* < 0.05 as the criteria. Finally, PICRUSt software was used to predict the functional profiles of rumen and fecal bacteria in the LALA and HALA groups based on the MetaCyc database (Caspi et al., 2020), and then STAMP software was employed to analyze the functional differences between the two groups (Liu et al., 2023).

Metabolomic data analysis was also conducted using R Studio. Principal component analysis (PCA) and orthogonal partial least squares-discriminant analysis (OPLS-DA) were performed using ropls package. PCA delineated global data distribution patterns, while supervised OPLS-DA constructed intergroup classification models. Differential metabolite selection was based on combined criteria, including variable importance in projection (VIP) scores >1.0, *p*-values < 0.05 from Student's *t*-tests, and fold change thresholds (|log_2_FC| >1), to ensure enhanced stringency. Metabolic pathway analysis was conducted via the MetaboAnalyst 6.0 platform (https://www.metaboanalyst.ca) (Pang et al., 2024), employing Fisher's exact test to evaluate pathway significance in the *Bos taurus* KEGG database. The visualization of the aforementioned images was performed using the ggplot2 package. Finally, differential microorganisms in rumen fluid and feces, as well as differential metabolites in rumen fluid and plasma that were enriched in KEGG pathways, were selected for Spearman correlation analysis. Correlations with |r| > 0.6 and *p* < 0.05 were considered to be significant.

## 3 Results

### 3.1 Effects of different ALA diets on apparent nutrient digestibility, rumen fatty acid composition, and serum indices in dairy cows

**Figure 1:**
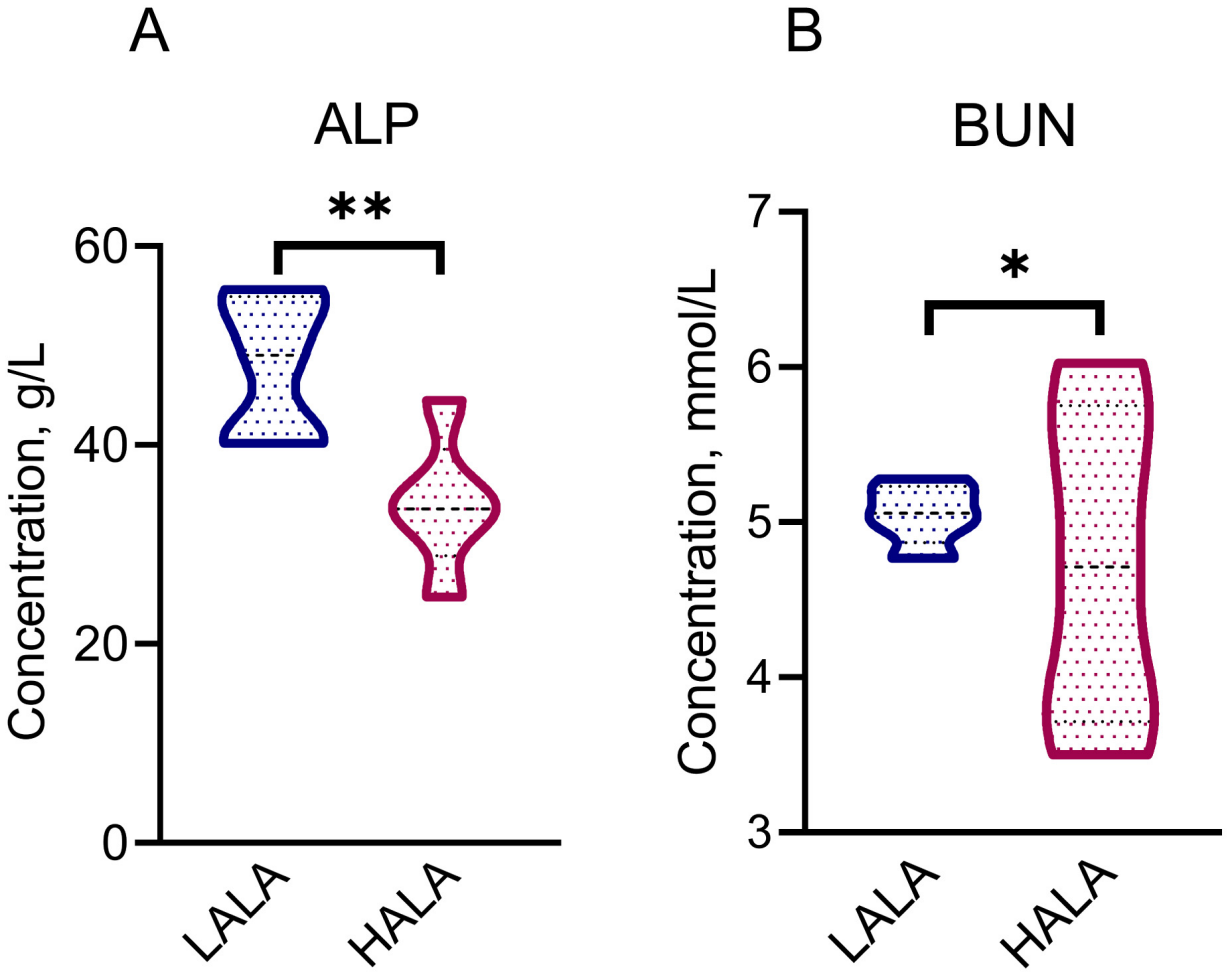
Differential serum physiological and biochemical indicators [ALP: **(A)**; BUN: **(B)**] between LALA and HALA groups. The statistical significance of the different treatments was assessed using Student's *t*-tests. *p < 0.05, **p < 0.01, ***p < 0.001. The absence of * indicated that the difference was not statistically significant.

Table 1 showed that the organic matter digestibility (OMD) in the HALA group was significantly lower than that in the LALA group (*p* < 0.05), whereas ether extract digestibility (EED) exhibited an increasing trend (*p* = 0.07). The effects of different proportions of ALA on rumen fatty acid composition were summarized in Table 2. Concentrations of C12:0, C14:0, C16:0, ΣSFA and the ratio of ω-6 unsaturated fatty acids (UFAs) to ω-3 UFAs were significantly higher in the LALA group compared to the HALA group (*p* < 0.05). Conversely, concentrations of C17:0, C18:0, C18:1n9c, C18:3n3, and ΣUFA were significantly higher in the HALA group (*p* < 0.05). Furthermore, results of serum biochemical and liver health indicators revealed that increasing the proportion of ALA in the diet significantly decreased the concentration of ALP (*p* < 0.01, Figure 1A). Additionally, BUN content in the HALA group was significantly lower than that in the LALA group (*p* < 0.05, Figure 1B). No significant differences were observed in other indicators (*p* > 0.05, Table 3).

**Table 1:** Apparent digestibility of nutrients in LALA and HALA groups of dairy cows.

**Items, %^1^**	**Groups** ^**2**^		**SEM**	***p*-value**
	**LALA**	**HALA**		
DMD	88.79	88.83	0.530	0.41
OMD	70.36^a^	69.38^b^	0.212	< 0.01
NDFD	53.51	49.62	1.588	0.25
ADFD	54.78	48.00	2.007	0.10
EED	64.74	72.66	2.207	0.07
CPD	70.13	68.43	0.854	0.36

^1^DMD, dry matter digestibility; OMD, organic matter digestibility; NDFD, neutral detergent fiber digestibility; ADFD, acid detergent fiber digestibility; EED, ether extract digestibility; CPD, crude protein digestibility.

^2^LALA, low proportion of ALA; HALA, high proportion of ALA.

The statistical significance of the different treatments was assessed using Student's *t*-tests and indicated by letters.

**Table 2:** Comparison of rumen fatty acid (FA) composition between LALA and HALA groups.

**Items, g/100 g of FA^1^**	**Groups** ^**2**^		**SEM**	***p*-value**
	**LALA**	**HALA**		
C10:0	0.15	0.14	0.011	0.61
C12:0	0.32^a^	0.25^b^	0.019	0.04
C13:0	0.13	0.11	0.017	0.54
C14:0	1.47^a^	0.87^b^	0.106	< 0.01
C15:0	1.00	0.94	0.050	0.62
C16:0	49.62^a^	19.80^b^	5.021	< 0.01
C17:0	0.67^b^	0.87^a^	0.044	0.01
C18:0	36.45^b^	60.22^a^	4.225	< 0.01
C18:1n9c	4.52^b^	9.82^a^	1.016	< 0.01
C18:2n6c	3.42	4.05	0.328	0.36
C18:3n3	0.73^b^	1.13^a^	0.093	0.02
C20:0	0.55	0.60	0.017	0.11
C20:1	0.28	0.46	0.050	0.06
C21:0	0.10	0.10	0.016	0.86
C22:0	0.22	0.22	0.030	0.93
C24:0	0.39	0.43	0.016	0.28
ΣSFA	91.06^a^	84.54^b^	1.402	< 0.01
ΣUFA	8.94^b^	15.46^a^	1.402	< 0.01
ω-6UFA/ω-3UFA	4.68^a^	3.57^b^	0.264	0.02

^1^ΣSFA, The content of the sum of saturated fatty acids; ΣUFA, The content of the sum of unsaturated fatty acids; ω-6UFA/ω-3UFA, the ratio of total ω-6 unsaturated fatty acids to ω-3 unsaturated fatty acids.

^2^LALA, low proportion of ALA; HALA, high proportion of ALA.

The statistical significance of the different treatments was assessed using Student's *t*-tests and indicated by letters.

**Table 3:** Comparison of serum biochemical indicators between LALA group and HALA group of dairy cows.

**Items^1^**	**Groups** ^**2**^		**SEM**	***p*-value**
	**LALA**	**HALA**		
GLU, mmol/L	4.25	4.28	0.049	0.82
TG, mmol/L	0.14	0.15	0.005	0.74
TC, mmol/L	5.75	5.30	0.224	0.32
NEFA, μmol/L	48.93	47.27	1.285	0.53
BHBA, mmol/L	0.39	0.40	0.015	0.86
INS, mIU/L	19.86	20.33	0.663	0.73
SOD, U/mL	40.43	39.64	1.128	0.73
T-AOC, U/mL	7.32	7.38	0.192	0.86
GSH-Px, μmol/L	3.06	3.03	0.105	0.88
TP, g/L	69.74	70.61	0.775	0.59
ALB, g/L	27.09	26.83	0.568	0.82
GLB, g/L	42.85	43.22	1.057	0.60
ALB/GLB	0.65	0.62	0.026	0.62
ALT, U/L	26.85	24.85	1.120	0.38
AST, U/L	82.71	73.99	2.712	0.11
T-BIL, μmol/L	6.82	6.61	0.187	0.57

^1^ALB/GLB, the ratio of albumin to globulin.

^2^LALA, low proportion of ALA; HALA, high proportion of ALA.

The statistical significance of the different treatments was assessed using Student's *t*-tests and indicated by letters.

### 3.2 Effects of different ALA diets on diversity, composition and differential bacteria of rumen bacteria, and fecal bacteria

**Figure 2:**
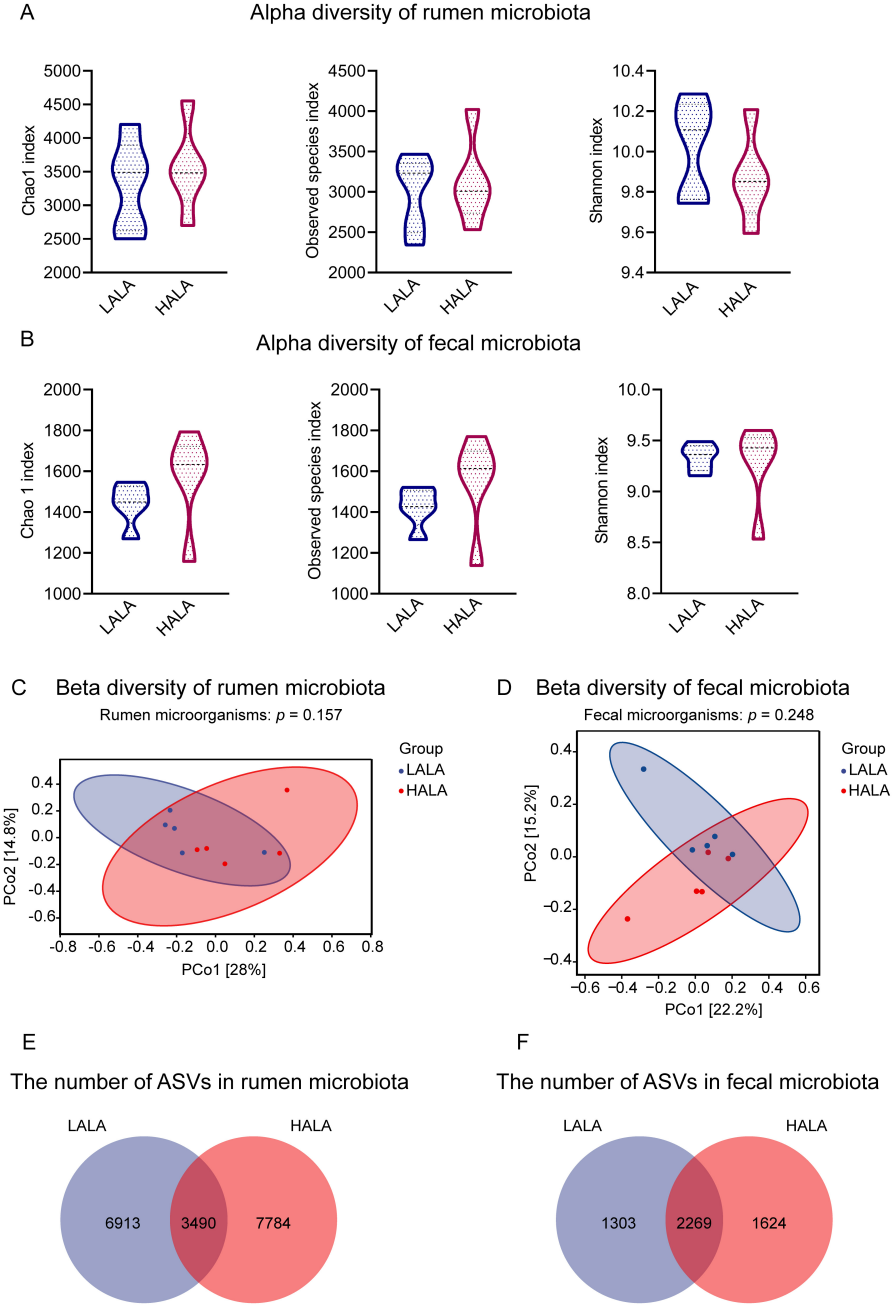
**(A, B)** Alpha diversity and richness of rumen and fecal microorganisms in the LALA and HALA groups. The alpha diversity indices evaluated included the Chao1 index, Observed species index, and Shannon index. **(C, D)** PCoA plots based on the Bray-Curtis distance matrix at the ASV level for rumen and fecal microorganisms, respectively. PERMANOVA results with 999 permutations were provided (rumen microorganisms: *p* = 0.157; fecal microorganisms: *p* = 0.248). The horizontal and vertical axes represented PCo1 and PCo2, respectively, indicating the proportion of variability they explain. **(E, F)** The number of shared and unique ASVs in the rumen microbial community and fecal microbial community. LALA, low proportion of ALA; HALA, high proportion of ALA. *p < 0.05, **p < 0.01, ***p < 0.001. The absence of * indicated that the difference was not statistically significant.

To investigate the alpha diversity of rumen and fecal microorganisms, the Chao 1 index, Observed species index and Shannon index were calculated. PCoA based on the Bray-Curtis distance was employed to assess the beta diversity of the microbial community. Results indicated no significant differences in alpha diversity or beta diversity of bacterial communities between the LALA and HALA groups in both rumen fluid and fecal samples (*p* > 0.05; Figures 2A–D). Venn diagram analysis revealed that in rumen fluid samples, 3,490 common ASVs were found, with 6,913 and 7,784 unique ASVs identified in the LALA and HALA groups, respectively (Figure 2E). In fecal samples, 2,269 common ASVs were detected, along with 1,303 and 1,624 unique ASVs in the LALA and HALA groups, respectively (Figure 2F).

**Figure 3:**
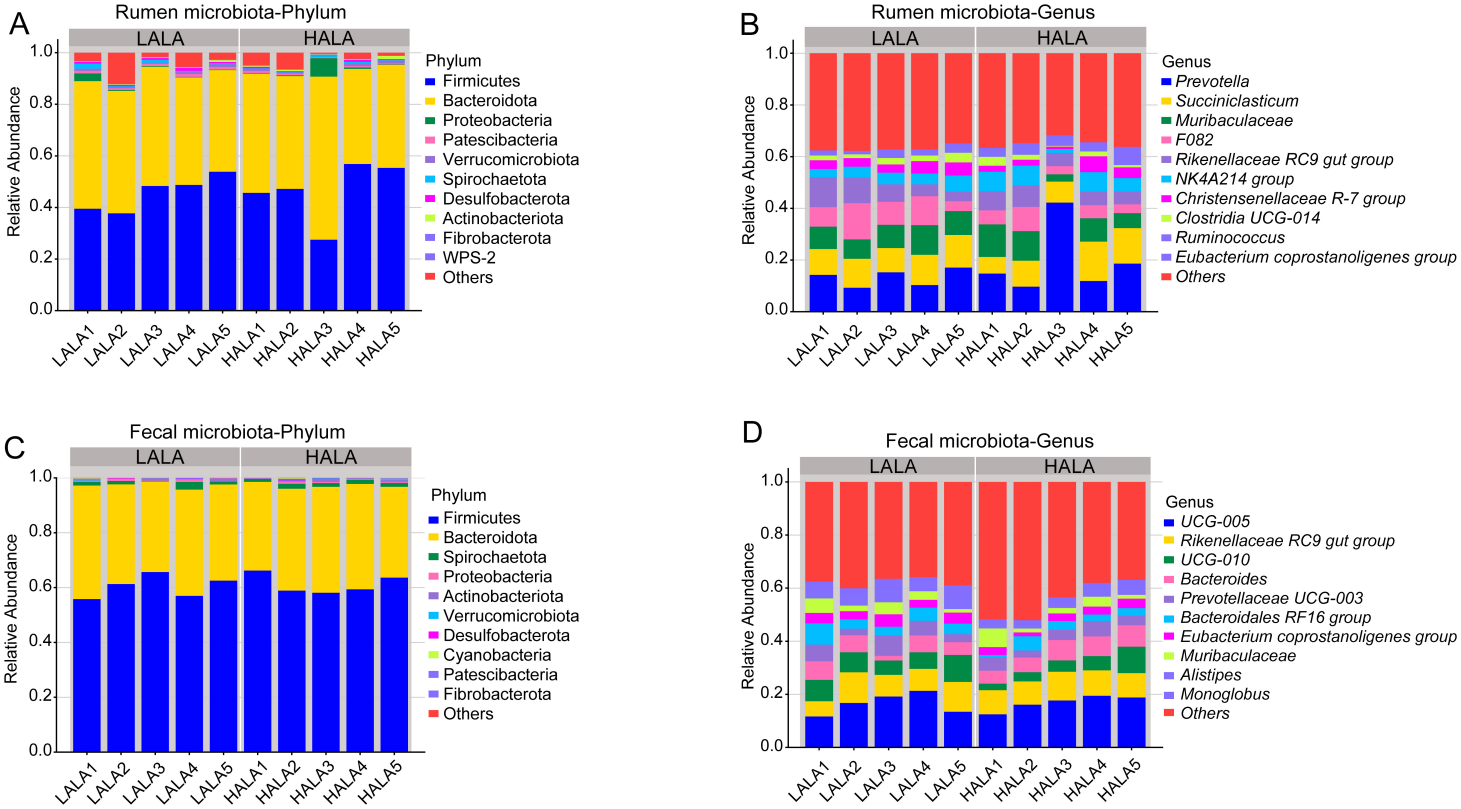
**(A, B)** The phylum-level and the genus-level composition of the top 10 rumen microbiota. **(C, D)** The phylum-level and the genus-level composition of the top 10 fecal microbiota.

To investigate the microbial composition of rumen and fecal microorganisms in LALA group and HALA group, the taxonomic composition of species was analyzed. Figure 3A showed that the dominant bacteria in the rumen of the two groups were Firmicutes (LALA: 45.66% ± 6.79%, HALA: 46.53% ± 11.70%) and Bacteroidota (LALA: 44.85% ± 4.18%, HALA: 46.01% ± 10.26%). Figure 3B showed that the top five relative abundances of rumen microbiota in the LALA group were *Prevotella* (13.26% ± 3.32%), *Succiniclasticum* (10.96% ± 1.30%), *Muribaculaceae* (9.19% ± 1.45%), *F082* (9.09% ± 3.85%) and *Rikenellaceae RC9 gut group* (7.28% ± 3.37%). In the HALA group, the top five relative abundances rumen microbiota were *Prevotella* (19.46% ± 13.17%), *Succiniclasticum* (10.71% ± 3.71%), *Muribaculaceae* (8.35% ± 4.05%), *Rikenellaceae RC9 gut group* (6.26% ± 1.53%), *NK4A214 group* (5.84% ± 2.63%). Similarly, Firmicutes (LALA: 60.47% ± 4.05%, HALA: 61.25% ± 3.52%) and Bacteroidota (LALA: 36.92% ± 3.25%, HALA: 35.94% ± 3.00%) were the dominant bacteria in the feces of both groups (Figure 3C). Figure 3D showed that the top five relative abundances fecal microbiota genera in the LALA group were *UCG-005* (16.49% ± 3.97%), *Rikenellaceae RC9 gut group* (8.98% ± 2.43%), *UCG-010* (7.49% ± 1.83%), *Bacteroides* (5.26% ± 2.13%), and *Prevotellaceae UCG-003* (4.98% ± 2.20%). In the HALA group, the top five relative abundances fecal microbiota genera were *UCG-005* (16.91% ± 2.79%), *Rikenellaceae RC9 gut group* (9.48% ± 0.81%), *Bacteroides* (6.70% ± 1.42%), *UCG-010* (5.12% ± 2.92%), and *Prevotellaceae UCG-003* (4.19% ± 1.24%).

**Figure 4:**
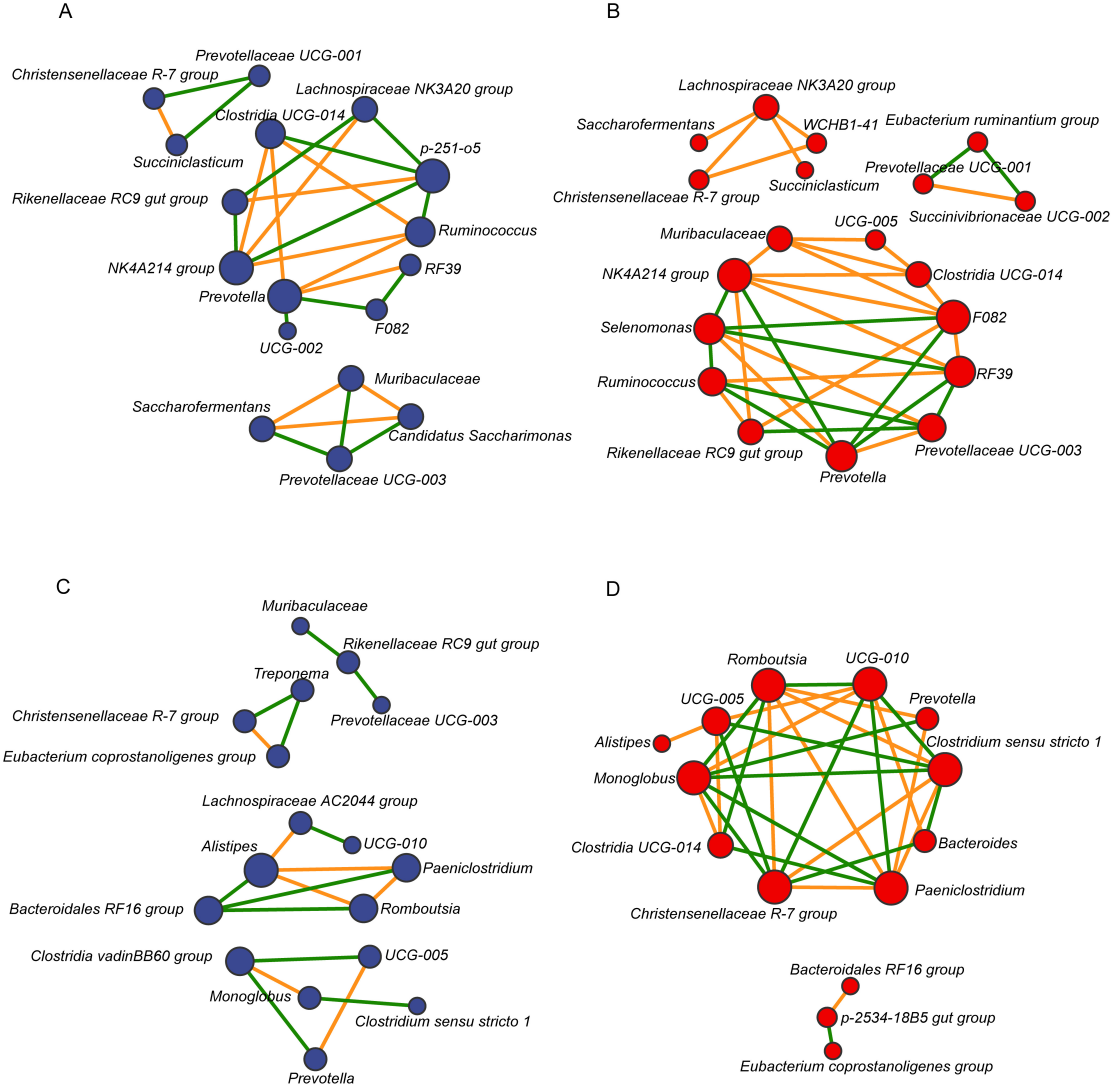
Spearman correlation networks of the top 20 most relatively abundant bacterial taxa in the rumen [LALA: **(A)**; HALA: **(B)**] and fecal [LALA: **(C)**; HALA: **(D)**] microbial communities. The blue nodes indicated genera in the LALA group and the red nodes indicated genera in the HALA group. The color edges stood for strong (Spearman's correlation coefficient < −0.6 or > 0.6) and significant (*p* < 0.05) correlations between genera, including positive correlations (orange) and negative correlations (green). LALA, low proportion of ALA; HALA, high proportion of ALA.

To explore interactions within the microbial community, a microbiome correlation network map was constructedusing the top 20 bacterial genera based on relative abundance in the rumen and feces of dairy cows from both groups. The results showed that the rumen microbial network of the LALA group comprised 17 nodes and 26 edges, of which 12 were positively correlated and 14 were negatively correlated (Figure 4A). The rumen microbiome network of the HALA group consisted of 19 nodes and 36 edges, with 23 positive and 13 negative correlations (Figure 4B). For fecal bacteria, the LALA group's network included 17 nodes and 18 edges (7 positive and 11 negative correlations, Figure 4C), while the HALA group's network had 14 nodes and 31 edges (15 positive and 16 negative correlations, Figure 4D).

**Figure 5:**
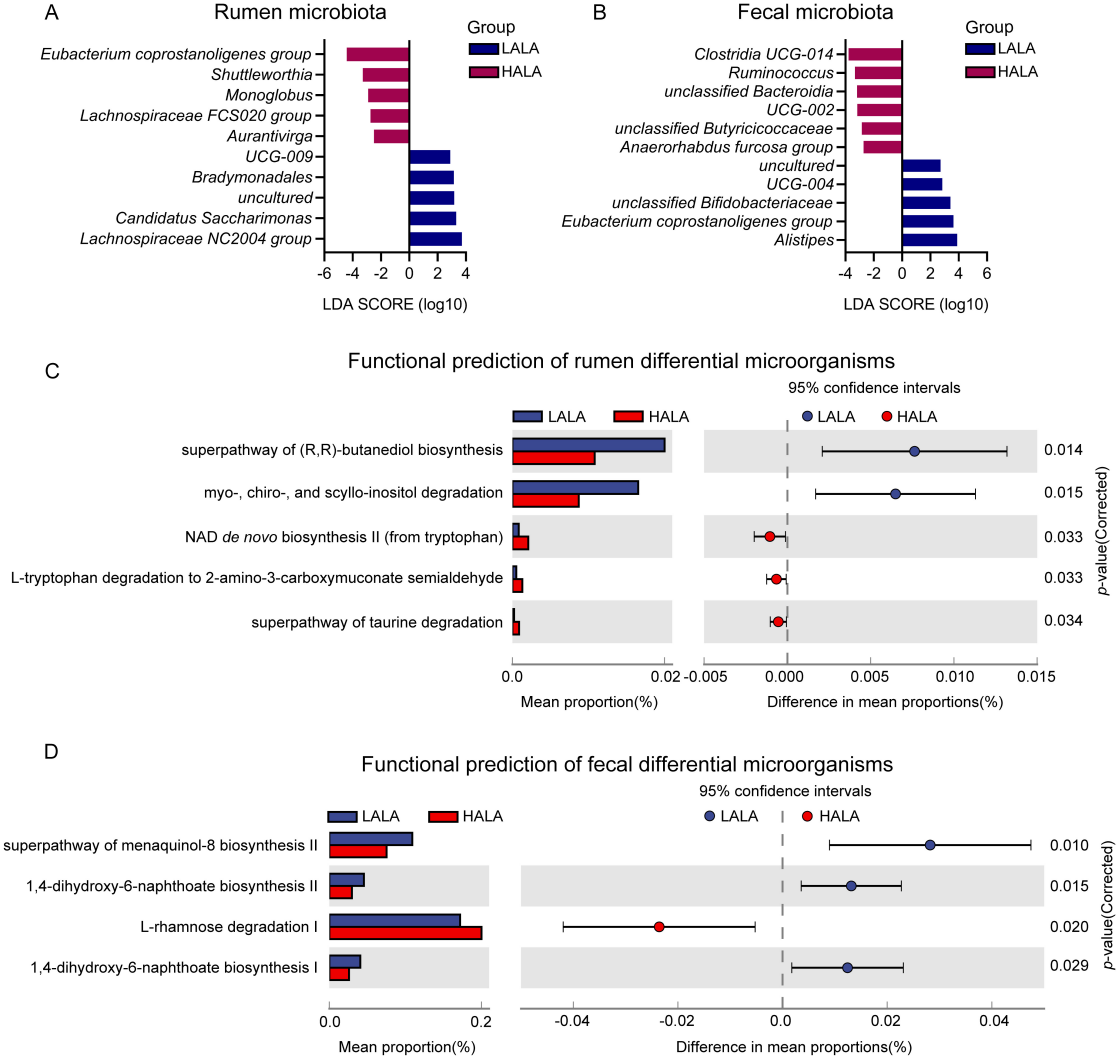
**(A, B)** The rumen differential microbiota and fecal differential microbiota based on LEfSe analysis in LALA and HALA groups. Significant differences were identified based on |LDA| > 2 with *p* < 0.05. **(C, D)** The functional differences of rumen microorganisms and fecal microorganisms in dairy cows from the LALA and HALA groups. PICRUSt software was used to predict the functional profiles of rumen and fecal bacteria in the LALA and HALA groups based on the MetaCyc database and then STAMP software was employed to analyze the functional differences between the two groups. LALA, low proportion of ALA; HALA, high proportion of ALA.

To identify inter-group variations in microbial communities, LEfSe was performed with an LDA score threshold of 2 and a *p*-value threshold of 0.05. The results indicated significant differences in rumen and fecal bacterial structure between the LALA and HALA groups. Figure 5A showed that in the LALA group, rumen bacteria with high LDA scores included *UCG-009, Bradymonadales, uncultured, Candidatus Saccharimonas* and *Lachnospiraceae NC2004 group*. In the HALA group, rumen bacteria with relatively high LDA scores were *Eubacterium coprostanoligenes group, Shuttleworthia, Monoglobus, Lachnospiraceae FCS020 group* and *Aurantivirga*. Moreover, Figure 5B showed that in feces, *uncultured, UCG-004, unclassified Bifidobacteriaceae, Eubacterium coprostanoligenes group*, and *Alistipes* had higher LDA values in the LALA group, while *Clostridia UCG-014, Ruminococcus, unclassified Bacteroidia, UCG-002, unclassified Butyricicoccaceae*, and *Anaerorhabdus furcosa group* were differential microbita in the HALA group.

To investigate the potential functional roles of these differentially abundant microbiota, microbial function prediction was performed. Figure 5C indicated that in the LALA group, differential microorganisms in the rumen were significantly associated with the superpathway of (R,R)-butanediol biosynthesis and myo-, chiro-, and scyllo-inositol degradation (*p* < 0.05). In the HALA group, rumen differential microorganisms showed significant changes in the NAD *de novo* biosynthesis II (from tryptophan), L-tryptophan degradation to 2-amino-3-carboxymuconate semialdehyde, and the superpathway of taurine degradation (*p* < 0.05). For differential microorganisms in the feces (Figure 5D), significant enrichment was observed in the pathways of 1,4-dihydroxy-6-naphthoate biosynthesis I and II, as well as the superpathway of menaquinol-8 biosynthesis II in the LALA group (*p* < 0.05). In the HALA group, fecal differential microorganisms were significantly enriched in the pathway of L-rhamnose degradation I (*p* < 0.05).

### 3.3 Effects of different ALA diets on rumen metabolites and plasma metabolites

**Figure 6:**
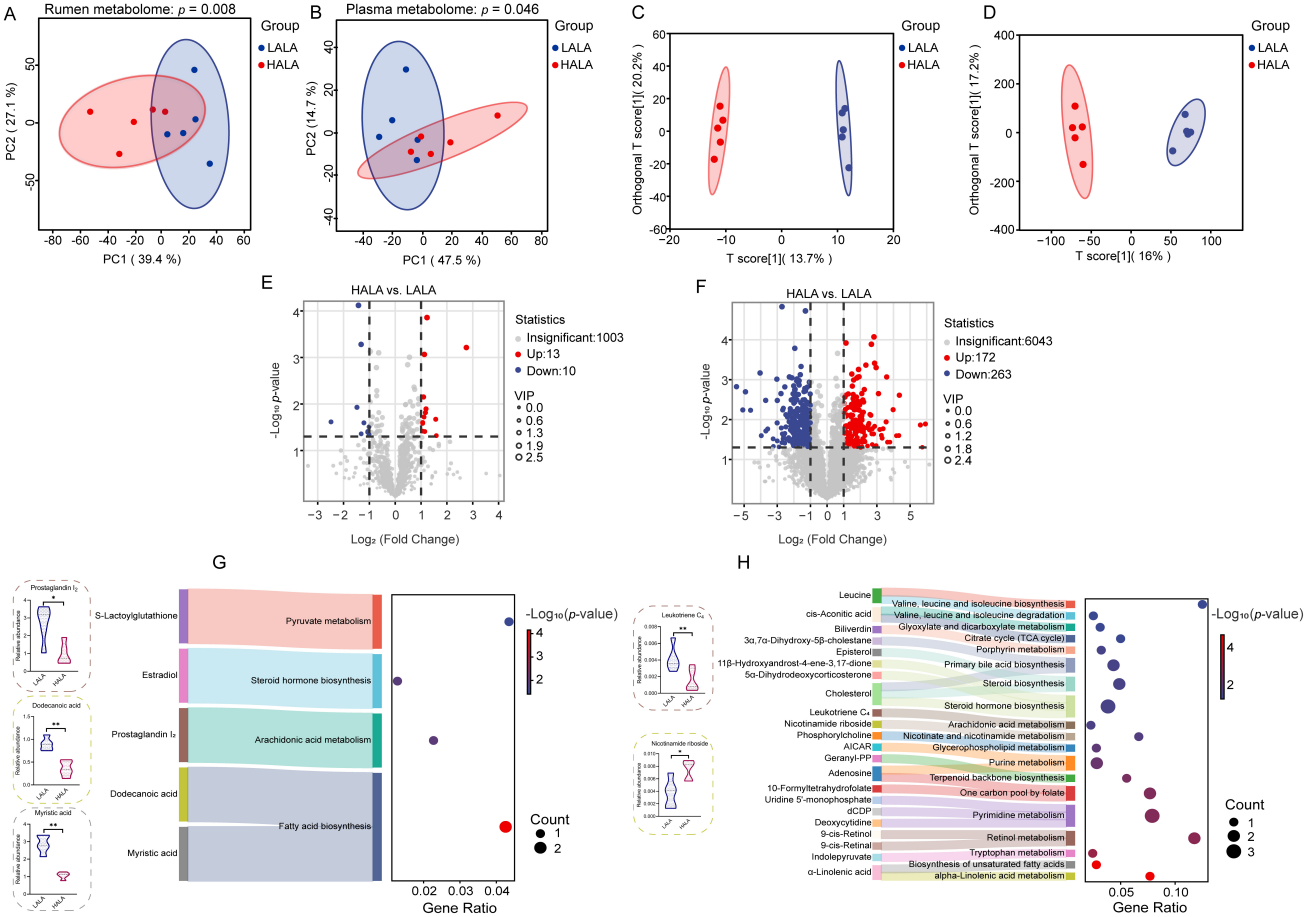
**(A, B)** PCA plots for the rumen metabolome and plasma metabolome, along with PERMANOVA results based on 999 permutations (rumen metabolome: *p* = 0.008; plasma metabolome: *p* = 0.046). The horizontal and vertical axes represented PC1 and PC2, respectively, indicating the proportion of variability they explained. **(C, D)** OPLS-DA models of the rumen metabolome and plasma metabolome. In these plots, the horizontal axis represented the T score of the first predictive principal component, while the vertical axis represented the Orthogonal T score. **(E, F)** The differential metabolites identified in the rumen and plasma metabolomic profiles. Significant differences were identified based on VIP scores >1.0, *p*-values < 0.05 from Student's *t*-tests, and fold change thresholds (|log_2_FC| >1). **(G, H)** The significantly enriched pathways of differential metabolites in the rumen and plasma, with the ratio representing the proportion of enriched metabolites relative to all metabolites in the pathway, and displayed the key differential metabolites concentrations in the rumen and plasma, respectively. Fisher's exact test was employed to evaluate pathway significance in the *Bos taurus* KEGG database. LALA, low proportion of ALA; HALA, high proportion of ALA. *p < 0.05, **p < 0.01, ***p < 0.001. The absence of * indicated that the difference was not statistically significant.

To visually examine the patterns and differences in metabolome data within the same dimension, PCA figures were initially performed on the metabolome data. The results of two PCA plots indicated significant separation between the LALA and HALA groups (*p* < 0.05, Figures 6A, B), and OPLS-DA of rumen and plasma metabolites revealed a clear distinction between the LALA and HALA groups (Figures 6C, D).

Differential metabolites were screened using VIP > 1 and *p* < 0.05 as criteria. Volcano plots of metabolites showed that a total of 1,026 metabolites were detected in the rumen fluid, with 23 differential metabolites (13 upregulated and 10 downregulated, Figure 6E). In plasma, 6,478 metabolites were detected, with 435 metabolites differentially regulated (172 upregulated and 263 downregulated, Figure 6F). Due to the complexity of metabolite names and types, some differential metabolites remained unidentified. In this study, 12 identified rumen fluid differential metabolites and 121 identified plasma differential metabolites were selected for KEGG enrichment analysis. The results indicated that differential metabolites in rumen fluid were significantly enriched in four key pathways: fatty acid biosynthesis, AA metabolism, steroid hormone biosynthesis, and pyruvate metabolism (Figure 6G). Notably, as the key differentially inflammatory-associated metabolites in rumen fluid, dodecanoic acid and myristic acid were enriched in the fatty acid biosynthesis, whereas prostaglandin I_2_ (PGI_2_) was enriched in the AA metabolism pathway, all of which were presented at lower concentrations in the HALA group compared to the LALA group. Differential metabolites in plasma were significantly enriched in 19 pathways, including ALA metabolism, AA metabolism, biosynthesis of unsaturated fatty acids, tryptophan metabolism, retinol metabolism, and pyrimidine metabolism (Figure 6H). In addition, as the key differentially inflammatory-associated metabolites in plasma, leukotriene C_4_ (LTC_4_) was also enriched in the AA metabolism pathway, whereas nicotinamide riboside (NR) was enriched in nicotinate and nicotinamide metabolism. Interestingly, the plasma differential metabolite LTC_4_ was presented at lower concentrations in the HALA group compared to the LALA group while NR was found at higher concentrations in the HALA group.

### 3.4 Significant correlations exist among differential rumen microbiota, rumen metabolites, plasma metabolites, and fecal microbiota

To further clarify the interplay between gastrointestinal microbiota and their metabolites across ALA treatments, we conducted correlation analyses among the rumen differential microbiota, fecal differential microbiota, rumen differential metabolites and plasma differential metabolites.

**Figure 7:**
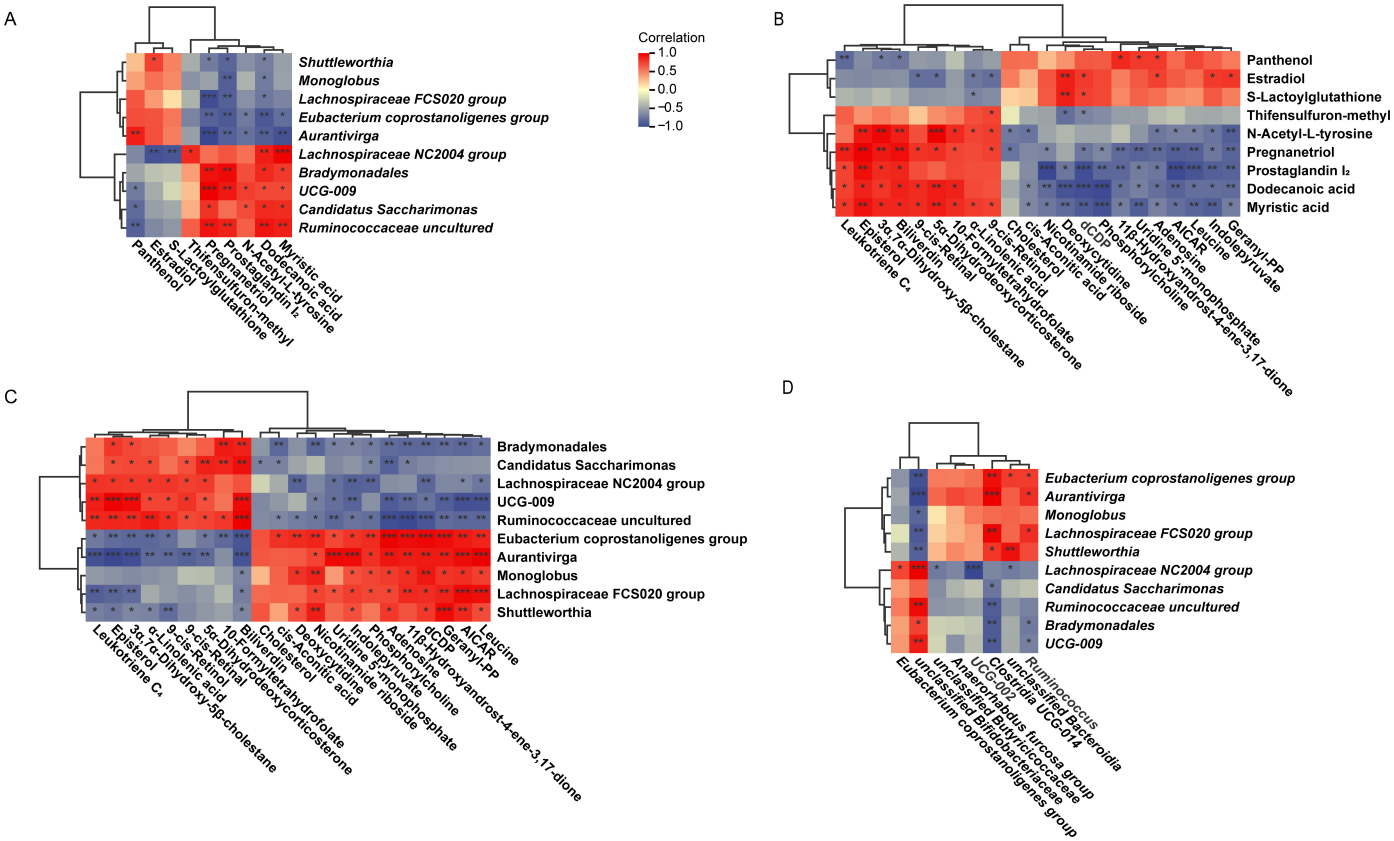
**(A)** Correlation analysis of rumen differential microbes and rumen differential metabolites. **(B)** Correlation analysis of rumen differential metabolites and plasma differential metabolites. **(C)** Correlation analysis of rumen differential microbes and plasma differential metabolites. **(D)** Correlation analysis of rumen differential microbes and fecal differential microbes. LALA, low proportion of ALA; HALA, high proportion of ALA. Spearman correlation analysis was performed, and correlations with |r| > 0.6 and *p* < 0.05 were considered statistically significant. *p < 0.05, **p < 0.01, ***p < 0.001. The absence of * indicated that the difference was not statistically significant.

Results showed that rumen differential microbiota in the HALA group were negatively associated with dodecanoic acid (*p* < 0.05), myristic acid and PGI_2_ (*p* < 0.05), whereas rumen differential microbiota in the LALA group displayed the opposite pattern (Figure 7A). These three rumen metabolites, in turn, were positively linked to the pro-inflammatory plasma mediator LTC_4_ (Figure 7B, *p* < 0.05). Consequently, rumen differential microbiota in the HALA group were negatively correlated with plasma LTC_4_, while LALA differential microbiota showed the inverse relationship (Figure 7C). Cross-compartment analysis further demonstrated that rumen differential microbiota in the HALA group were positively correlated with their own fecal differential microbiota but negatively correlated with those of LALA cows, with LALA rumen microbiota exhibiting the reverse configuration (Figure 7D).

## 4 Discussion

In dairy cows, dietary UFAs not only serve as an energy source but also exert regulatory effects on immune function and gut microbial ecology (Jawhara, 2023; Liu et al., 2025; Sun et al., 2022). Previous studies have demonstrated that polyunsaturated fatty acids (PUFAs), particularly ALA, can modulate the composition and activity of the gastrointestinal microbiota, thereby influencing host metabolism and immune responses (Zhang et al., 2017; Zhu L. et al., 2020). However, the precise mechanisms by which varying levels of dietary ALA regulate immunity via the gut microbiota and its metabolites remain poorly understood. To address this gap, we conducted a controlled feeding trial integrating 16S rRNA gene sequencing and metabolomic analyses. Our aim was to investigate how different dietary ALA levels affect host immune function by reshaping the gastrointestinal microbial ecosystem. Our results demonstrate that ALA supplementation modulates key microbial taxa, thereby influencing metabolites involved in inflammatory response regulation and ultimately enhancing immune function in dairy cows. These findings provide valuable insights for optimizing ALA inclusion in dairy cow diets to modulate metabolic pathways and potentially promote animal health.

The ATTD in dairy cows reflects the efficiency of nutrient utilization (Hao et al., 2020). Previous studies have indicated that the effects of ALA on dairy cow ATTD are nutrient-specific (Martin et al., 2008; Pi et al., 2019). One study reported that feeding dairy cows extruded flaxseed diets decreased whole-tract OMD (Martin et al., 2008), whereas another demonstrated that diets enriched in ALA via flaxseed oil significantly increased the ATTD of EE (Pi et al., 2019). Both outcomes are consistent with our study. Furthermore, this study analyzed the fatty acid composition of rumen contents collected via oral cannulation from dairy cows fed diets with varying levels of dietary ALA, without filtration. Although this approach didn't differentiate between solid-phase and liquid-associated fatty acids and might underestimate total fatty acid flux relative to whole digesta analysis, it offered a practical and high-temporal-resolution method for evaluating rapid, early-phase metabolic responses in the rumen following dietary intervention. A significant increase in ALA and other UFAs was observed in the rumen fluid of cows in the HALA group; however, whether and how these proximal ruminal metabolic alterations influence the quality traits of the final product remains central to assessing their biological significance. Notably, a parallel study conducted by our research team using the same cohort of experimental animals provided essential terminal phenotypic data (Yang et al., 2024). That study demonstrated that the identical HALA dietary intervention—corresponding to the HUFA group (and the identical LALA dietary intervention—corresponding to the LUFA group) in the referenced work—significantly increased milk n-3 PUFA content, including ALA, and effectively reduced the n-6/n-3 PUFA ratio. Integrated findings from both studies suggest that diet-induced changes in the ruminal fatty acid profile at this primary site of digestion are likely propagated through absorption and systemic metabolism, ultimately contributing to improved milk fat composition. This integration not only provides downstream functional validation for the present rumen fluid analysis but also establishes a coherent mechanistic pathway—from dietary intervention to initial ruminal metabolic responses and onward to enhanced dairy product quality—thereby strengthening the scientific robustness and practical relevance of this work. Serum ALP primarily originates from the liver and bones and serves as an indicator of hepatobiliary system pathology (Esbrit et al., 1975; Prokopič and Beuers, 2021), commonly used in assessing hepatic function. Notably, a significant increase in ALP concentration is often observed in dairy cows with liver impairment (Sun et al., 2021). In this study, the ALP concentration in the HALA group was significantly lower than that in the LALA group, suggesting that increasing dietary ALA content may have a positive impact on the health status of dairy cows.

It is increasingly evident that the composition of mammalian gut microbial communities is significantly influenced by dietary factors (Ross et al., 2024). These microbiota establish complex mutualistic relationships with their hosts, which have substantial implications for overall health (Ley et al., 2008). Therefore, perturbations in the composition or functionality of the gut microbiota may play a critical role in the development of health conditions associated with metabolic alterations (Boulangé et al., 2016). In this study, the rumen and fecal microbiota were affected by altering the proportion of ALA. Notably, compared with the LALA group, we found the relative abundance of *Eubacterium coprostanoligenes group* in rumen was higher in the HALA group. *Eubacterium coprostanoligenes group*, a member of the phylum Firmicutes, possesses strong potential for sterol transformation and fatty acid regulation (Freier et al., 1994). Furthermore, accumulating evidence indicates that this microbiota can promote intestinal mucus secretion, thereby enhancing the integrity of the intestinal mucus barrier, resisting pathogenic microbial invasion, and reducing inflammatory responses (Bai et al., 2024). Supplementing flaxseed in the diet has been shown to significantly increase the relative abundance of *Eubacterium coprostanoligenes group* in the rumen (Huang et al., 2021), which is consistent with our results. These results indicate that high levels of ALA have the potential to modulate host metabolism by altering the composition and function of the rumen microbiota. Fecal microbiota analysis revealed that the relative abundance of *Ruminococcus* and *Clostridia UCG-014* were significantly higher in the HALA group compared to the LALA group. Both *Ruminococcus* and *Clostridia UCG-014* belong to the phylum Firmicutes. *Ruminococcus* present in feces is capable of fermenting a variety of complex carbohydrates, including cellulose, pectin, and resistant starch (Walker et al., 2011; Ze et al., 2012), and serve as key producers of acetate and propionate (Christopherson et al., 2014; Crost et al., 2013). Meanwhile, *Clostridia UCG-014*, also detected in fecal microbiota, contributes to the production of short-chain fatty acids (SCFAs) (Quraishi et al., 2022). These SCFAs play a critical role in maintaining intestinal homeostasis by modulating host immune responses and inflammatory pathways (Akhtar et al., 2021). Furthermore, studies have demonstrated that the relative abundance of Clostridia UCG-014 is positively associated with levels of tryptophan metabolites—compounds shown to possess protective effects against colitis—indicating its potential involvement in preserving gut microbial equilibrium and supporting host health (Feng et al., 2025; Yang et al., 2021b). Therefore, this study demonstrates that a high dietary proportion of ALA increases the relative abundance of gastrointestinal beneficial fermentative bacteria, thereby enhancing the host's anti-inflammatory response.

Gut microbiota play a crucial role in maintaining metabolic homeostasis, with their functional activities primarily manifested through the metabolites they produce, which contribute to the regulation of host metabolic balance (Meng et al., 2020). Previous studies have demonstrated that microbial metabolites play a pivotal role in modulating the host's immune response (Campbell et al., 2023; Fujisaka et al., 2018). Specifically, among the differential metabolites identified in rumen fluid, dodecanoic acid, a saturated 12-carbon medium-chain fatty acid, is a marker of impaired liver function and inflammatory response in the gut (Luo et al., 2024). Myristic acid, a saturated 14-carbon fatty acid with oral activity, has been shown to increase adipose inflammation and insulin resistance associated with systemic obesity in mice on a high-fat diet (Saraswathi et al., 2022). Therefore, it is hypothesized that the increased abundance of *Eubacterium coprostanoligenes group* in this study may have reduced the accumulation of medium-chain saturated fatty acids, like dodecanoic acid and myristic acid, in the rumen through enhanced microbial utilization of fatty acid. Another important metabolite, PGI_2_, is an AA metabolite generated via the cyclooxygenase pathway (Norlander et al., 2021). After feeding fish oil rich in ALA, the concentration of AA in the gastric and duodenal tissues decrease, and the synthesis of prostaglandins is reduced (de la Hunt et al., 1988). In this study, the reduced concentration of PGI_2_ in rumen fluid was consistent with findings from previous studies. Potential explanations for this reduction may include the redirection of cellular fatty acid metabolic pathways by ALA, which reduces AA metabolism and consequently decreases the production of prostaglandin-like substances (de la Hunt et al., 1988).

Host blood metabolites, functioning as sensitive biomarkers, reflect dynamic physiological states and environmental exposure profiles, serving as crucial indicators for monitoring metabolic pathway activities and evaluating environmental impacts (Lee et al., 1978). NR, a precursor to NAD (Nicotinamide Adenine Dinucleotide) and a source of niacin, reduces the expression of inflammation and glycolysis genes by activating the AMPK-SIRT1/SIRT3 pathway (Cantó et al., 2012). This finding aligns with the predictive functions of gastrointestinal microbiota with differences in relative abundance—NAD *de novo* biosynthesis II (from tryptophan). These results support the hypothesis that the intestinal microbiota contributes to the biosynthesis of NR via the NAD^+^ precursor metabolic pathway. Another metabolite with a reduced concentration is LTC_4_. LTC_4_ is a member of the cysteinyl leukotrienes (CysLTs) family and plays a critical role in the pathophysiological processes associated with various inflammatory responses and diseases (Amat et al., 1998; Evans, 2005). Existing studies demonstrate that LTC_4_ promotes the secretion of β-glucuronidase by macrophages, thereby exacerbating inflammatory responses (Schenkelaars and Bonta, 1986), and being strongly associated with the severity of systemic inflammation (Zipser et al., 1987). Previous study have shown that increasing the proportion of ALA in the diet can reduce the formation of leukotrienes derived from AA in polymorphonuclear leukocytes, which may help alleviate the severity of LTs-mediated allergic and inflammatory responses (Hashimoto et al., 1988). The observed decrease in LTC_4_ concentration in the host serum in this study indicates that dietary supplementation with a high proportion of ALA may exert potential anti-inflammatory effects on the host. Therefore, feeding a high proportion of ALA not only improves the rumen and posterior intestinal microbial community structure in dairy cows but also may enhance the host's ability to resist inflammatory responses by modulating the metabolic responses in the host.

Microbial metabolites not only accumulate within the gastrointestinal tract but are also actively absorbed and utilized by host tissues, leading to the induction of both metabolic and immune responses in the host (Uchimura et al., 2018). In this study, a comprehensive analysis of metabolic pathways in rumen fluid and plasma revealed that the AA metabolism and steroid hormone biosynthesis pathways were common to both metabolomics datasets. AA, an important PUFA, is primarily metabolized through the COX pathway, LOX pathway, and cytochrome P450 (CYP) pathway (Hanna and Hafez, 2018). The inhibition of the AA metabolic pathway leads to a decrease in inflammatory mediators, including prostaglandins, leukotrienes, and lipid derivatives, thereby mitigating the inflammatory response (Wang et al., 2021), and this finding is consistent with the observed reduction in PGI_2_ and LTC_4_ concentrations in our results. These findings suggest that a high dietary intake of ALA may suppress AA metabolism in both microbial communities and the host, potentially conferring beneficial effects on overall health.

**Figure 8:**
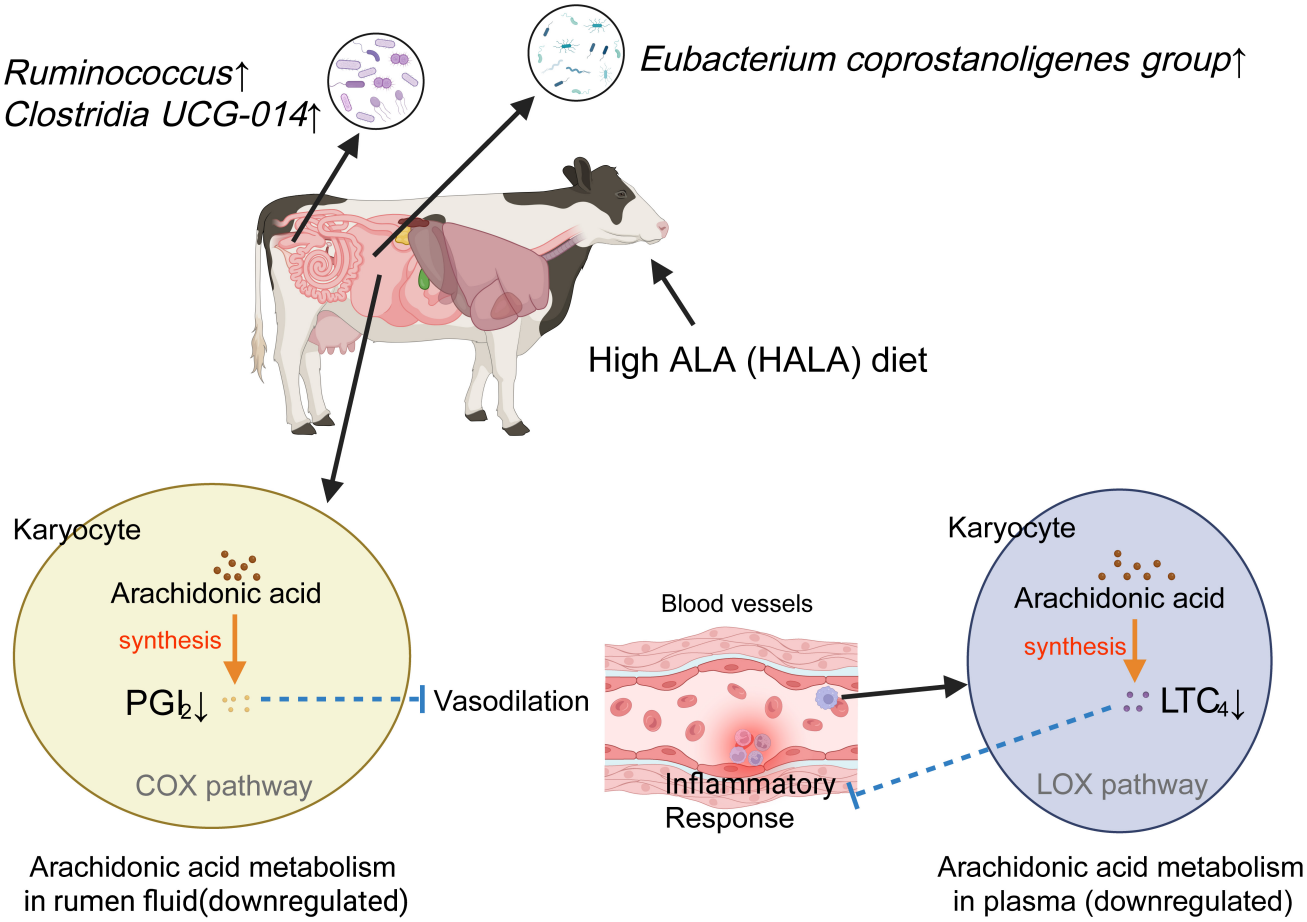
Anti-inflammatory mechanism of high proportion of ALA diet. Solid lines represented experimentally observed biological processes within the metabolic pathway, whereas dashed lines indicated putative or hypothetical pathways. Arrows denote stimulatory effects in the direction of biosynthesis, while lines terminated by perpendicular bars signify inhibitory regulation. PGI_2_, Prostaglandin I_2_; COX, Cyclooxygenase_;_ LTC_4_, Leukotriene C_4_; LOX, Lipoxygenase. Image created with BioRender.com, with permission.

Interestingly, correlation analysis revealed that the rumen differential microbial taxon—*Eubacterium coprostanoligenes group*—in the HALA group was negatively associated with the down-regulated rumen metabolite PGI_2_ and the down-regulated plasma metabolite LTC_4_, and positively correlated with the upregulated fecal differential microbial taxa—*Clostridia UCG-014* and *Ruminococcus*. In this study, correlation analysis between the rumen and plasma differential metabolites revealed that PGI_2_ was positively correlated with LTC_4_ in the HALA group. Moreover, they were also significantly enriched in the AA metabolism pathway. The proposed mechanistic pathway linking dietary ALA to immunomodulation via the gut microbiota and metabolite changes was visually summarized in Figure 8. Our findings demonstrate that a high dietary inclusion of ALA suppresses the production of inflammatory mediators in dairy cow plasma by inhibiting AA metabolism in both rumen microorganisms and the host. In the subsequent phase of this study, metagenomic data will be systematically analyzed to elucidate the specific mechanisms through which ALA modulates the inflammatory response via microbial metabolites. Furthermore, targeted validation experiments will be conducted to reinforce these findings and establish a solid scientific foundation for the application of ALA in dairy cow diets.

## 5 Conclusions

This study systematically investigated the effects of ALA on the microbiota and metabolite profiles of gastrointestinal tract, and its interactions with host metabolism in dairy cows, using an integrated approach combining 16S rRNA gene analysis with non-targeted metabolomics. The results demonstrate that a high proportion of dietary ALA can reshape the rumen and fecal microbial community structure and may enhance immune function by inhibiting the microbial-mediated AA metabolic pathway, thereby reducing the inflammatory response in dairy cows. However, this study included only two ALA levels (low and high), which limits the ability to establish a dose-response relationship or identify the optimal dosage. Overall, our findings provide a scientific basis for optimizing ALA levels in dairy cow diets to improve host health, although the underlying mechanisms linking ALA, gut microbiota composition, and immune responses require further investigation.

## Data Availability

The original contributions presented in the study are publicly available. The 16S rRNA gene sequence data that support the findings of this study have been deposited into the national center for biotechnology information (NCBI) Sequence Read Archive (SRA) under the accession number PRJNA1295766 and PRJNA1295848.
